# Near-Field Electrospinning and Melt Electrowriting of Biomedical Polymers—Progress and Limitations

**DOI:** 10.3390/polym13071097

**Published:** 2021-03-30

**Authors:** William E. King, Gary L. Bowlin

**Affiliations:** 1 Department of Biomedical Engineering, University of Memphis, Memphis, TN 38152, USA; wking14@uthsc.edu; 2 Department of Biomedical Engineering, University of Tennessee Health Science Center, Memphis, TN 38163, USA

**Keywords:** near-field electrospinning, melt electrowrite, fiber write, biomedical polymer

## Abstract

Near-field electrospinning (NFES) and melt electrowriting (MEW) are the process of extruding a fiber due to the force exerted by an electric field and collecting the fiber before bending instabilities occur. When paired with precise relative motion between the polymer source and the collector, a fiber can be directly written as dictated by preprogrammed geometry. As a result, this precise fiber control results in another dimension of scaffold tailorability for biomedical applications. In this review, biomedically relevant polymers that to date have manufactured fibers by NFES/MEW are explored and the present limitations in direct fiber writing of standardization in published setup details, fiber write throughput, and increased ease in the creation of complex scaffold geometries are discussed.

## 1. Near-Field Electrospinning (NFES)/Melt Electrowriting (MEW): Fiber Writing

Intentional build design and material choice are paramount for any successful biomedical scaffold applications. To date, scaffold manufacturing techniques such as freeze-drying, solvent casting with particle leaching, gas foaming, and traditional electrospinning (TES) have all been used for tissue engineering (TE), drug delivery, and microelectromechanical systems (MEMS) applications [1]. Of these manufacturing processes, electrospinning, both solution and melt, have gained considerable popularity due to its robust variety of polymer options and consistent throughput. These processes principally consist of a charged, flowable polymer and a counter electrode. The subsequent electric field exerts a force on the flowable polymer to form a Taylor cone. When the force exceeds the surface tension of the solution/melt polymer, a liquid jet is extruded and accelerated towards the counter electrode. As the melt cools or the solvent evaporates in the air gap, a fiber is formed, while simultaneously incurring Rayleigh axisymmetric, electric field-induced axisymmetric, and bending instabilities [2,3]. These instabilities chaotically whip the fiber until it is randomly deposited on the collecting surface. Unfortunately, the random deposition of fibers does limit scaffold design possibilities and thus the techniques of near-field electrospinning (NFES) and melt electrowriting (MEW) were devised as a solution to the chaotic fiber.

In 2003, Kameoka et al. demonstrated a method of controlling individual electrospun fibers by reducing the air gap distance and provide relative motion between the polymer spinneret and collector before bending stabilities can occur [4]. When paired with precise translational movement between the polymer source and collector, fibers can be directly “written” allowing for the creation of specific geometries that can be laid down layer-by-layer. Thus, introducing another dimension of scaffold tailorability to create custom programmed geometries independent of processing parameters. As a result, architectures with open interconnected or closed pores and perfectly aligned fibers or pseudorandom fibers are all possible with one manufacturing technique.

As NFES, MEW, and TES all utilize a Taylor cone formed in an electric field, it suggests that if a polymer solution/melt has the viscosity, conductivity, and surface tension for extrusion by TES, then fiber extrusion by NFES should also possible. Numerous polymers have been successfully TES for biomedical applications and the continued exploration of these polymers through direct fiber writing is of extreme interest [5]. In this review, the current progress of NFES/MEW processed biomedical polymers is detailed and prominent limitations of standardization in published setup details, fiber write throughput, and increased ease in the creation of complex scaffold geometries are discussed.

## 2. Fiber Writing of Biomedical Polymers

### 2.1. Alginate

**Figure 1:**
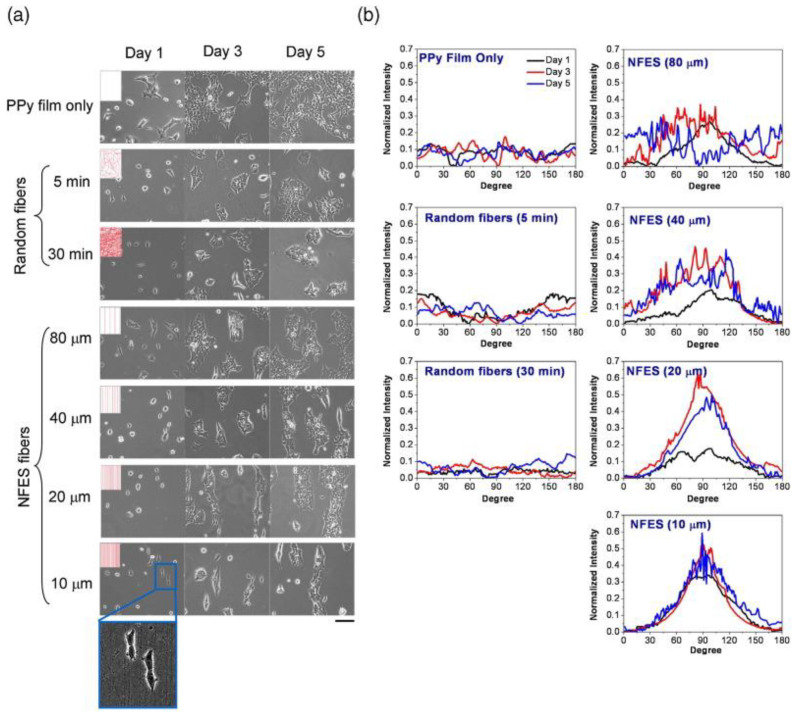
Cell alignment on near-field electrospinning (NFES) fibers. (**a**) HEK 293T cells were seeded to PPy films, randomly deposited alginate fibers, or NFES alginate fibers deposited surfaces. The distribution and morphology of surface cells were observed by a phase contrast microscope (scale bar = 100 μm). (**b**) Cell images were analyzed by fast Fourier transform (FFT) to determine the levels of cell alignment. Reproduced with permission from Fuh et al., Materials Science and Engineering: C; published by Elsevier, 2016.

Alginates are polysaccharides consisting of (1 to >4) linked β-d-mannuronate and alpha-l-guluronate. This biopolymer is harvested from brown seaweed and when exposed to divalent cations such as Ca^2+^ form hydrogels, which have numerous biomedical applications including drug delivery and tissue engineering [6]. In 2016, Fuh et al. were the first to explore alginate’s capacity for aligned NFES fibers to modify a biomaterial’s surface in order to dictate cell patterning [7]. Specifically, stock solutions of 5% alginate and 5% poly(ethylene oxide) (PEO) in deionized water were prepared to extrude NFES fibers onto polypyrrole (PPy) films using a programmable X-Y stage. Fiber diameter was explored over the processing parameters of applied voltage (0.7–1 kV), air gap distance (0.5–2 mm), and alginate concentration (2–3.5 wt %) using a 22 gauge (G) needle. The resulting fibers were reported with an average fiber diameter ranging from 0.4 to 0.9 µm, and also, fiber placement precision was evaluated for parallel fibers spaced at 10, 20, 40, and 80 µm. Bright-field images were qualitatively evaluated and showed consistent fiber placement. The authors crosslinked the 10, 20, 40, and 80 µm spaced alginate fibers with 2% CaCl_2_ for 2 h followed by seeding human embryonic kidney cells (HEK 293T) on the NFES manufactured alginate fibers, TES manufactured alginate fibers, and PPy film for 1, 3, and 5 days, Figure 1a–b. At each time point, phase-contrast images were gathered and the cell body alignment was evaluated using the fast Fourier transform (FFT) of the images. No trends in cell alignment were observed at all time points from TES fabricated alginate fibers and PPy films. NFES fibers resulted in sharp peaks of cell alignment at 10 and 20 µm fiber spacing at all time points, but 40 and 80 µm spaced alginate fibers showed considerably less alignment. Qualitative images showed that HEK cells did not readily adhere to the alginate fibers and were being loosely bound in between fibers to achieve alignment. At 3 days, HEK cell viability was quantified using an MTT assay. There was a significant reduction from 85% in 80 µm spaced fibers to 75% for 10 µm spaced in relative viability compared to PPy film. The authors hypothesized that tailoring the alginate fiber degradation by reducing the CaCl_2_ crosslinking concentration would result in better cell proliferation. HEK cells were subsequently seeded on 10 µm spaced alginate fibers crosslinking with 0, 0.5, 1, or 2% CaCl_2_ for 1, 3, and 5 days. All crosslinked fibers showed sharp alignment at all time points with non-crosslinked fibers showing a consistent but reduced alignment at all time points. Cell relative viability showed a linear trend with a significant difference of 70% in 2% crosslinked fibers compared to 90% in uncrosslinked fibers. Thus, the authors demonstrated that alginate solutions could be processed by NFES to produce uniform, defect-free microfibers on the surface of a biomaterial and that these fibers can dictate cell patterning. Nevertheless, this work is still in the early proof-of-concept phase as only 2D parallel fibers were evaluated for potential future work with cell alignment and biocompatibility. Their NFES setup did allow for uniform fiber placement as close as 10 µm but ideally, their methodology needed to include spinneret/collector translational velocity, spinneret needle length, and the polymer flow rate in addition to the applied voltage, air gap, and needle gauge to be completely reproducible.

### 2.2. Poly(γ-benzyl-l-glutamate) (PBLG)

PBLG is a synthetic polypeptide whose molecular structure consists of α -helices in organic solvents. When electrospun, the α -helices are highly aligned making the material processes by electrospinning ideal for biocompatible piezoelectric applications [8]. In 2014, Pan et al. were the first to explore the electrical energy conversion and mechanical characteristics of piezoelectric fibers of PBLG processed by NFES for potential applications as energy harvesters [9,10]. Specifically, PBLG with a molecular weight of 48,822 g/mole (g/mol) was dissolved in dichloromethane to reliably produce microfibers using their custom X-Y table with a rotating cylindrical mandrel collector. Aligned PBLG fibers were deposited orthogonal to the axis of the mandrel’s rotation to evaluate the processing parameters of solution concentration (10–20 wt %), polymer solution flow rate (1–3 mL/h), spinneret needle diameter (0.15–0.25 mm), translational velocity (524–3142 mm/s), and applied electric field (2 × 10^6^–1.5 × 10^7^ V/m). The authors reported that fibers between 2.5 and 44 µm could be created and that there was an inverse relationship between fiber diameter and solution concentration and electric field strength. Strips of aligned fiber constructs were cut to 1 mm × 15 mm and mechanically evaluated in tension until failure. Testing showed a Young’ modulus of 3.6 GPa and ultimate tensile strength (UTS) of 60.5 MPa (reported without standard deviations) compared to poly(vinylidene difluoride) (PVDF) created from the same apparatus with values of 0.8 GPa and 37.9 MPa, respectively. The piezoelectric properties of their PBLG fibers were evaluated by creating a Thévenin equivalents circuit with 20 mm × 15 mm sections of the material being subjected to strains of 0.05% at 18.3 Hz. These data showed a maximum voltage of 33 mV was achieved at an external load resistance of 8 MΩ, which resulted in a power output of 138.4 pW. The authors then extruded NFES processed PBLG fibers onto cicada wings to harvest energy from their beating motion. The wings were artificially vibrated from 10 to 30 Hz with the maximum power occurring at the greatest wing vibration frequency of 30 Hz to produce a measured voltage of 14.3 mV and 19 nA of current. In summary, this work leverages the NFES process to create PBLG microfibers that achieve aligned α-helices suitable for piezoelectric energy harvesting applications and to fiber write complex geometries for biomedically relevant applications. The lack of reported standard deviations and statistics relegates NFES manufacture of PBLG fibers as a highly promising proof of concept but paves a way for future work with physiologic specific energy harvesting geometries.

### 2.3. Poly(ε-caprolactone) (PCL)

PCL is a hydrophobic, semicrystalline polymer with a relatively low melting point of 60 °C and is soluble in many organic solvents. Consequently, these properties result in PCL being easily manipulated into a variety of forms to be suitable for both solution and melt electrospinning. In vivo, its degradation mechanism occurs over 1–3 years through hydrolytic cleavage of its poly(α-hydroxy) ester bond [11]. As a result, PCL is currently the most prolific polymer used for fiber writing and has the most mature development for biomedical applications. Towards its mature development, in 2017 Delalat et al. MEW constructed PCL scaffolds to evaluate their capacity as T cell expansion platforms with expedited cell recovery for bench to bedside therapies [12]. A custom-built MEW setup was used to create PCL fibers with optimized parameters of 90–100 °C melt temperature, 23 G spinneret, 10 µm/hr flow rate, applied voltage of 10.5–12.5 kV, and an air gap of 20 mm to produce fibers of 13 ± 0.43 µm. Grid spacings of 200, 500, and 1000 µm were used to create scaffolds measuring 120–150 mm^2^ and an optimized 10 layers deep. As a comparison, scaffolds were created using the same MEW setup with a programmed pseudo-random geometry. Scaffold surfaces were functionalized by coating in an allyl glycidyl ether polymer that was subjected to a plasma polymerization process. The functionalized scaffolds were incubated in anti-human CD3 and CD28 antibodies ranging from 10 to 80 µg/mL to covalent conjugation to the surface. Antibody-bound scaffolds were cultured with density gradient purified CD4^+^ cells for 7 days and compared to the commercial product CD3/CD28 Human T-Activator Dynabeads. It was reported that the 200 µm spaced grids resulted in the greatest cell output per scaffold with an average of 1.1 × 10^6^ cells compared to the 1000 µm spaced scaffolds with 0.6 × 10^6^ cells. The authors demonstrated that the 200 µm spaced grids were the densest scaffold, resulting in the largest surface area to volume to 0.329 µm^−1^, and that this allowed for the most cell/scaffold interactions per volume. Furthermore, T cell expansion was found to be significantly greater in the organized scaffolds compared to the random geometry with a total CD4^+^ scaffold output of 2.4 × 10^6^ cells compared to 5 × 10^5^ cells, but there was no significant difference in CD4^+^ cell output for MEW PCL scaffolds and Dynabead expansion at approximately 17 fold each. While there was no increase in expansion over the current gold standard, the lack of beads in the PCL scaffold resulted in one less processing step to remove the beads before using the cells therapeutically. This would further increase the therapy’s safety as there would be no chance of persistent beads reaching the patient. Subsequently, the authors isolated CD25^+^ CD127^dim^ Treg cells from the CD4^+^ positive pool using fluorescence-activated cell sorting (FACS). After expansion and rest, FOXP3 and CH25 fluorescent staining were significantly higher at approximately double in MEW PCL lattices compared to Dynabeads. The authors concluded that this platform could efficiently expand stable populations of T cells without the need for further purification and thus easier cell retrieval compared to the current gold standard. Thus, this work demonstrated a non-load bearing scaffold application for MEW, and the tailorability afforded by PCL direct fiber writing resulted in a high surface area and pore volume for cell expansion applications. As a result, it is anticipated that this platform will have numerous future uses in research and industrial T cell expansion. Regarding reproducible methodology, it would have been ideal if the authors included their optimized translational velocity and spinneret needle length in their parameters.

**Figure 2:**
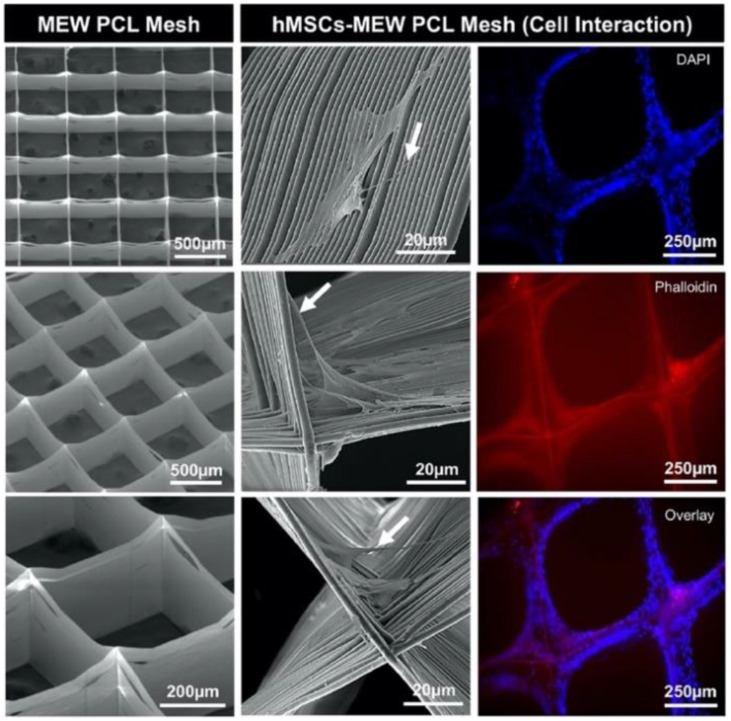
(**Left**) Representative SEM micrographs of melt electrowriting (MEW) PCL mesh show the well-aligned (0–90°-oriented junctions) fibrous 3D architecture with a 500 μm pore size and a mean fiber diameter of 3.16 μm. (**Middle**) SEM micrographs of hMSCs-MEW PCL mesh interaction after 3 days of culture. Note significant cell attachment, proliferation, and protrusion along and around the printed PCL fibers. Filopodia are also indicated (white arrows). (**Right**) Fluorescence staining of hMSCs-MEW PCL mesh interaction after 3 days showing phalloidin (Red) staining of filamentous actin and DAPI (Blue) for the nucleus (for interpretation of the references to color in this figure legend, the reader is referred to the web version of this article). Reproduced with permission from Dubey, N et al., Acta Biomaterialia; published by Acta Materialia, Inc., 2020.

A tissue engineering and regeneration application of written PCL fibers was demonstrated in 2020, by Dubey et al. who MEW manufactured PCL scaffolds to evaluate their ability to reinforce GelMA hydrogels and improve the composite material’s osteogenic ability for guided bone regeneration [13]. A 3DDiscovery system by regenHU was used to MEW extrude PCL fibers at optimized parameters of 90 °C melt temperature, 0.07 MPa feed pressure, 26 G spinneret, −7 kV applied voltage, 4 mm air gap, and 40 mm/s translational speed to produce fibers with a mean diameter of 3.16 µm. Three-dimensional scaffolds architecture was created with 500 µm spaced grid geometry for 450 layers. PCL scaffolds were placed in a silicone mold and filled with 20% GelMA laden with 0, 2.5, or 5% amorphous magnesium phosphate (AMP) followed by photocrosslinking. Hydrogels and PCL reinforced hydrogels were mechanically evaluated in compression at a strain rate of 0.5 mm/min. The GelMA hydrogels alone had a compressive stiffness of approximately 70 kPa compared to the statistically greater 315 kPa of the GelMA hydrogels reinforced with 4 PCL scaffolds. In vitro analysis of hydrogels with and without PCL scaffolds for all AMP concentrations were evaluated with seeded human mesenchymal stem cells (hMSCs), Figure 2. Cell proliferation by an MTS assay showed that hydrogels with PCL scaffolds containing 2.5 and 5% AMP had significantly greater proliferation at days 1 and 3 compared to GelMA hydrogel alone. On day 5, PCL reinforced hydrogels with 5% AMP had greater proliferation than GelMA with 5% AMP alone. Mineralization potential was evaluated using Alizarin red staining. At both 14 and 21 days, PCL reinforced hydrogels with 5% AMP had statistically greater mineralization than all other groups. Real-time PCR was used to evaluate relative fold changes in osteogenic gene expression of RUNX2, COL1, and OPN. At 21 days, PCL reinforced hydrogels with 5% AMP had statistically greater expression of all three osteogenic genes compared to other groups and a 4–5 fold increase over GelMA alone. In vivo evaluation was conducted with 6 weeks old male Fischer 344 rats. A 5 mm critical-sized bilateral calvarial defect was administered and treated with either a sham, GelMA, GelaMA with PCL scaffold, GelMA with 5% AMP, or GelMA with PCL scaffold and 5% AMP. At 4 and 8 weeks, rats were euthanized and their calvarias harvested to evaluate for bone formation by micro-CT and histology. At both time points, GelMA with PCL scaffold and 5% AMP had the statistically greatest bone volume as measured by micro-CT of 6 and 8 fold increase compared to the sham group. Hematoxylin and eosin staining, and Mason trichrome staining, reinforced these results, as at 4 weeks the sham group showed fibrous connective tissue and minimal bone at the edges, while the GelMA with PCL scaffold and 5% AMP showed newly formed bone that was well integrated at the edges and regenerated collagen tissue. In summary, this study demonstrated the feasibility of a commercially available biomedical MEW platform to produce high-quality 3D PCL scaffolds to significantly improve bone tissue regeneration. It is anticipated that future works include evaluating these scaffolds under dynamic load conditions both in vitro and in vivo. Nevertheless, it would have been ideal if the authors had also compared melt electrospun random PCL fibers in GelMA hydrogels to better demonstrate the advantages of MEW.

### 2.4. Chitosan

Chitosan is a linear polysaccharide derived from the alkalinization of chitin from crustaceans and is comprised of β-(1–>4) linked acetylated and deacetylated D-glucosamine units [14]. Fuh et al. were the first to explore in 2012 the NFES fabrication of chitosan fibers for the creation of a cell-based research platform for studying cell adhesion, spreading, and tissue architecture [15]. Chitosan with 85% deacetylation was dissolved in 0.5 M acetic acid to make a 4% solution were then mixed in a 90/10 or 50/50 ratio with a 5% PEO solution followed by the addition of unreported volumes of 0.5 wt % Triton-X 100 and 5–10 wt % dimethylsulphoxide (DMSO). The chitosan solutions could reliably produce NFES fibers using a programmable X-Y stage to make a variety of geometries including parallel lines, concentric curves, and grids. The fiber diameter was explored for the 50/50 blend chitosan/PEO blend using a translational velocity of 20 cm/s, polymer flow rate of 0.1 µL/h, and 0.4 mm inner diameter (ID) spinneret needle over the range of processing parameters of applied voltage (0.6–1.2 kV) and air gap distance (0.5–1.5 mm). It was shown that fibers could be created between 0.27 and 1.3 µm and using identical optimized processing parameters, the authors showed that 90/10 chitosan/PEO resulted in an average fiber diameter of 0.8 ± 0.1 µm compared to 0.4 ± 0.1 µm for a 50/50 blend. In subsequent studies, Fuh et al. explored the capacity for chitosan/PEO fibers to dictate cell morphology and spreading [16]. In this work, they used a 5% chitosan solution with 1% PEO dissolved in 0.5 M acetic acid. The authors’ optimal parameter of 0.5 mm air gap, 0.8 kV applied voltage, and 200 mm/s translational velocity were used to created consistent fibers with an average diameter of 0.72 µm. Chitosan fibers were then deposited onto polypyrrole (PPy) modified tissue culture plastic (TCP) first in parallel lines with 20 and 100 µm spacing followed by grids with 20 and 100 µm spacing. HEK cells were seeded for 1 and 3 days with bright-field microscopy images taken at each time point. ImageJ was used to perform a fast Fourier transform (FFT) analysis of the HEK cellular alignment on aligned chitosan fibers, traditionally electrospun chitosan fibers, and PPy covered TCP. The authors reported a sharp alignment of 90° for HEK cells seeded on parallel fibers at both spacings. Qualitatively, the cells were bounded in between the chitosan fibers and adhered to the PPy surface, and also, there were no discernable trends observed in the grid geometry for chitosan fibers and TES fibers and PPy surface. The HEK cells did not adhere and align with the chitosan/PEO fibers on a PPy substrate like with NFES manufactured alginate fibers and alternatively behaved as cells in a trough. In conclusion, the authors showed that chitosan can be reliably processed by NFES to create consistent cylindrical fibers in a 2D plane but did not explore the capacity for 3D scaffold creation. Furthermore, this particular chitosan fiber composition in the context of a PPy substrate should not be considered for an integrative tissue engineering strategy as cells preferred to be bound between fibers. It is anticipated that future works for this platform to be used as a tool to study cell adhesion and spreading.

### 2.5. Collagen

Collagen is the primary extracellular matrix (ECM) structural protein and is comprised of three tropocollagen molecules wound in a right-handed triple helix. Collagen has extensive applications in tissue engineering as recapitulating the ECM with the native material is a promising strategy [17]. In 2019, Alexander Jr. et al. were the first to demonstrate that type I bovine collagen could be processed via NFES by characterizing the range of processing parameters to ultimately create scaffolds to mimic native ECM [18]. The authors dissolved the type I collagen at a concentration of 250 mg/mL in 40–60% acetic acid could be extruded by NFES using an X-Y stage to provide the precise relative motion for fibers to be deposited onto gold-coated glass slides. The system was enclosed in a temperature and humidity-controlled chamber and was driven by a custom-built LabVIEW program to control the voltage supply, syringe pump, and stage movement. Holding air gap at 0.58 mm and spinneret needle gauge at 27 G constant, the authors evaluated the quality of fibers produced (defined as the number of straight lines of fibers produced versus lines attempted) based on acetic acid solvent percent (40–60%), relative humidity (RH) (20–50%), and applied voltage (1–1.5 kV). Bright-field images of fibers were acquired and qualitatively evaluated using a custom ImageJ and MATLAB program. The authors reported that acetic acid percent concentration and RH contributed the most to linear, cylindrical fiber formation with solvent concentrations of 45–50% and humidity less than 30% being the most ideal conditions for their setup. Collagen fiber mats consisting of 30 layers of fibers with 15 µm spacing were cut into 2.1 mm × 1.8 mm strips to be mechanically evaluated in tension. At this stage of the study, collagen mats were also evaluated using the apparatus to produce collagen fibers dissolved in hexafluoro-2-propanol (HFP) at a concentration of 138 mg/mL. Scaffolds were mechanically elongated until failure to ascertain Young’s modulus and load at failure. The results showed that collagen fibers from HFP solvent resulted in statistically greater load at failure of 1.1 N compared to acetic acid with 0.2 N. There was no statistical difference in Young’s modulus between the two solvents of approximately 0.3 MPa. In summary, the authors demonstrate that collagen could be extruded by NFES and that their custom apparatus setup was designed to control the minor processing parameters of RH and temperature to improve better consistency in fiber production. While these minor variables were studied, for this work to be reproduced, the authors needed to report translational velocity and polymer flow rate in their methodology. Additionally, it is important to note that NFES processed collagen fibers never exceeded a success rate of 66%. Therefore, further exploration in processing parameters and NFES setup will need to be explored to improve the success rate for this important ECM component.

### 2.6. Copolymers

Copolymers are derived from more than one type of monomer and the order of the individual types of monomers can range from individually alternating, to blocks of alternating monomer, and randomly distributed blocks. In 2015, Chen et al. were the first to investigate MEW processing of the copolymer poly(l-lactide-co-ε-caprolactone -co-acryloyl carbonate) (poly(LLA-ε-CL-AC)) [19]. This tripolymer consisted of the monomers ε-caprolactone, L-lactide, and acryloyl carbonate in a (25:65:10) ratio, which the authors hypothesized that this copolymer when crosslinked would be resistant to creep upon hydration for tissue engineering applications. After synthesis, the polymer was combined with Irgacure 651 photoinitiator to result in a 1% dry amount and was subsequently processed by MEW using a custom setup with processing parameters of 145 °C extrusion temperature, 3.0 bar extrusion gas pressure, 7.0 kV applied voltage, 30 G spinneret, 4.5 mm air gap, and a translational velocity of 7 mm/s. Grid scaffolds created were comprised of 24.6 ± 2.7 µm diameter fibers with 100 µm spacing stacked 20 layers deep. After completion, the scaffolds were subjected to UV light until cured followed by water absorption evaluation at 24 h and 7 days for both crosslinked and uncrosslinked fibers. At both time points, the uncrosslinked polymer had significantly more absorbed water with 15% and 20% at 24 h and 7 days compared to crosslinked at 3% and 6%, respectively. The scaffolds were then mechanically evaluated by uniaxial elongation until failure at a strain rate of 1%/s under dry and hydrated conditions and dynamically evaluated at 10% strain and 1 Hz for 10,000 cycles at physiological body temperature. The average modulus per fiber within each scaffold was then calculated with the data showing that hydrated and crosslinked fibers had an average modulus of 370 MPa, which persisted after 10,000 cycles. The uncrosslinked fibers had a significantly lower modulus of 35 MPa, which dropped to 13 MPa after 10,000 cycles. Thus, this work demonstrates a two-step process where the precrosslinked polymer is amenable to high-quality MEW fiber writing, and the desired mechanical properties can be achieved through crosslinking post scaffold creation. Anticipated future works for MEW of this polymer include scaffolds for tissue engineering of soft connective tissues.

Subsequently in 2018, Hochleitner et al. were the first to manufacture MEW fibers of the copolymer poly(urea-siloxane), a thermoplastic elastomer, for potential applications in bone tissue engineering [20]. This AB copolymer consisted of polydimethylsiloxane (PDMS) as the “soft” segment and urea as the “hard” segment. In this work, PDMS-diamine (DMS-A12) was reacted with 1,6-hexamethylene diisocyanate (HDMI) in tetrahydrofuran at 20 wt % solid to form poly(urea-siloxane), DMS-A12-HMDI. A custom MEW apparatus was used to evaluate the poly(urea-siloxane) over the range of processing parameters of heating temperature (80–100 °C), feeding pressure (1–3 bar), and applied voltage (8–12 kV) using a 24 G needle at an 8.5 mm air gap. For each evaluated parameter the critical translational speed (CTS), defined as the minimum velocity to achieve straight fibers, was determined and ranged from 2000 to 3250 mm/min. The authors reported well-formed fibers with a diameter range of 10.6 ± 0.6 to 17.8 ± 0.7 µm. Fibers were subsequently stacked into 1 mm spaced, 100 layer grids with perfect fiber placement. Scaffolds were mechanically evaluated in tension at room temperature conditions until failure. The Young’s modulus was determined to be 27.3 ± 1.6 MPa with stress and strain at failure being 2.04 ± 0.02 MPa and 773 ± 9%, respectively. The thermal stability of the poly(urea-siloxane) elastomer was evaluated to determine degradation under the temperature conditions required for MEW. The data showed that there was no change in melt viscosity at 90 °C for at least 14 h, which was 2–3 times the typical manufacturing time for their MEW scaffolds thus indicating a stable process. In conclusion, the authors demonstrated the first example of MEW fiber writing of a thermoplastic elastomer copolymer, and this copolymer platform is expected to have numerous promising applications for the creation of highly ductile biomedical scaffolds.

### 2.7. Polydioxanone (PDO)

PDO is a widely used, bioresorbable suture material clinically labeled as PDS II. PDO’s degradation mechanism occurs by hydrolysis of its ester bonds with an average in vivo degradation rate of 6 months [21,22]. In 2019, we were the first to explore the potential of NFES fabricated PDO fibers for tissue engineering applications as templates to guide cell alignment [23]. A commercially available 3D printer similar to Fettahi et al. was modified to provide the precise relative motion required for NFES. Exploring the range of processing parameters of air gap (1.8–3 mm), polymer concentration (140–220 mg/mL), spinneret gauge (18–23 G, 2 inches), translational velocity (10–100 mm/s), and applied voltage (1.1–1.6 kV) resulted in tailorable fibers diameters between 3.2 and 25 µm. Our results demonstrated that greater translational velocities resulted in a more consistent fiber diameter with a reduction of half the average standard deviation between 10 and 100 mm/s. Increasing translational velocity also results in an average increase in fiber crystallinity of up to 4% in PDO between 10 and 50 mm/s. The use of a commercially available 3D printer resulted in sufficient precision and accuracy to stack up to 80 fibers perfectly in a straight line ribbon and 30 layers in a grid geometry with mild off-target fiber placement. Lastly, we demonstrated that PDO templates with grid geometry were able to align gingival fibroblasts actin cytoskeletons using contact guidance with sharp alignment at 0° and ±90°. This was compared to the absence in the measured alignment of the fibroblast actin cytoskeleton in traditional electrospun PDO, membrane cast PDO, and tissue culture plastic. Our work demonstrated that NFES extrusion of PDO can produce high-quality fibers and that a modified, hobbyist 3D print has sufficient precision to stack PDO fibers to form 3D biomedical scaffolds. Future works are anticipated to include the creation of PDO scaffolds to serve as templates to guide in situ tissue regeneration. Furthermore, we are one of the few groups that report spinneret needle length and the gauge. This parameter is important as it has ramifications on fiber write stability and fiber size due to distribution of charge and thus should be consistently reported [24,25,26,27,28,29].

### 2.8. Polyethylene Oxide (PEO)

PEO is the higher molecular weight form (M_w_ > 10,000 g/mol) of polyethylene glycol (PEG) and is a widely used polymer with applications in hydrogel formation and as an excipient for pharmaceuticals [30]. PEO was used by Kameoka et al. during the first credited example of reducing the air gap distance of a charged polymer solution paired with a grounded collector and relative motion to directly write a fiber in 2003 [4]. In this work, the authors explored PEO dissolved in a 50/50 solution of DI water and ethanol. The setup consisted of a custom-made arrow-shaped silicon top that was coated in gold. In an “ink and quill” approach, the tip was dipped in the PEO solution, followed by translating the charged tip over a wafer counter electrode. The major processing parameters of solution concentration (5–30%), air gap distance (5–15 mm), translational velocity (168 cm/s), and applied voltage (4–6 kV) were investigated. The authors reported fiber diameter trends for PEO were proportional to polymer concentration and applied voltage and inversely proportional to air gap distance of translational velocity. Fiber diameters ranged from 100 to 1800 nm but the lack of a continual polymer supply resulted in fiber deposition limited to 5–10 s. In conclusion, Kamoeoka’s scanning tip electrospinning work was the founding example of electrofiber writing. While the ink and quill approaching served as an excellent proof of concept, it was not until a year later that the addition of a continuous polymer source by Sundaray et al. that this technique became viable for scaffold creation [31].

### 2.9. Poly(2-ethyl-2-oxazine) (PEtOzi)

**Figure 3:**
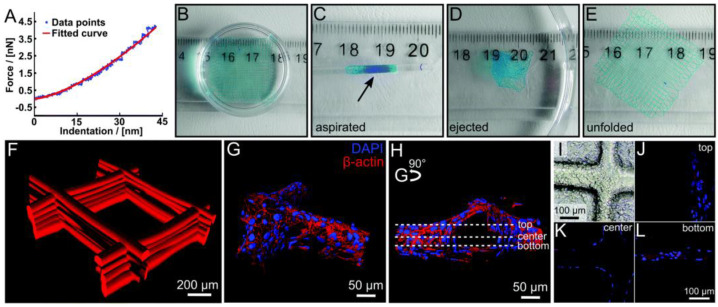
(**A**) Example of a force–distance curve for a swollen scaffold with a fitting curve using the Hertz model. (**B**–**E**) Illustration of the robust character of the hydrogel scaffolds. The swollen scaffolds (**B**) can be aspirated in a glass pipette (inner diameter 1.35 mm) (**C**) and ejected again repeatedly (**D**) without any visual damage to the scaffold, which unfolds (**E**) again by itself. Scaffolds were functionalized with DY-647P1-maleimide for better visualization. (**F**) Confocal microscope image of a fluorescently labeled hydrogel scaffold with 500 mm fiber spacing. (**G**–**L**) HEK293 cells were grown on hydrogel scaffolds functionalized with peptide (NH2-CGGGRGDS-COOH). (**G**,**H**) Cells were stained with cytoskeletal marker protein b-actin (red) and DAPI (nucleus, blue). Three-dimensional surface-reconstruction of HEK293 cells attached to scaffold (Imaris 7.6 software). (**I**) Phase-contrast image of seeded scaffold with HEK293 cells are attached along and around the scaffolds. (**J**–**L**) Representative images of one hydrogel filament within the whole scaffold from bottom, center, and top are shown, positions marked in (**H**). Video 3 (ESI†) provides a demonstration of aspiration and ejection, while Video 4 (ESI†) shows (G,H) in greater detail. Reproduced with permission from D. Nahm, F. Weigl, N. Schaefer, A. Sancho, A. Frank, J. Groll, C. Villmann, H. Schmidt, P. D. Dalton and R. Luxenhofer, Mater. Horiz., 2020, 7, 928-Published by The Royal Society of Chemistry. doi:10.1039/C9MH01654F.

PEtOzi is a hydrophilic polymer that is capable of spontaneous chemical crosslinking by a thermally reversible Diel-Alders reaction. In 2020, Nahm et al. were the first to explore the use of PEtOzi extrusion by MEW to directly write self-crosslinking hydrogels as the fibers cool post extrusion [32]. As a proof of concept, the authors demonstrated that 10 layers of precisely stacked fibers spaced 500 µm apart could be printed to form a directly written hydrogel scaffold in approximately 7 min of print time. Using optimized parameters of 2–4 kV applied voltage, 150 °C heated nozzle, and 1.5–2 bar polymer pressure, the dry MEW fibers measured 45 ± 5 µm in diameter and upon hydration, the fibers swelled to 89 ± 12 µm. The polymer platform was thermally responsive with an average 10% change in swelling area for every 10 °C. Atomic force microscopy (AFM) was used to evaluate the Young’s modulus of the hydrogel and was measured to be between 0.14 and 0.2 MPa, Figure 3A. These fiber-written hydrogels were mechanically robust to manipulation as the authors demonstrated that the PEtOzi scaffolds could be aspirated in a pipette and then ejected without unduly damaging the construct, Figure 3B–E. Biocompatibility of bulk PEtOzi was evaluated using a WST-1 elution assay with L929 mouse fibroblasts, and the data showed that cell viability after 48 h was >86% ± 3%. Subsequently, HEK cells were seeded onto the PEtOzi scaffolds and were evaluated after 4 days of culture. The cell nuclei and actin cytoskeleton were fluorescently stained and imaged by confocal microscopy, which the authors qualitatively reported that the HEK cells readily adhered to the hydrogel scaffold, Figure 3F–L. In summary, this work detailed the ability to fiber write a custom geometry, robust to handling hydrogel in less than 10 min, which has numerous promising biomedical applications. Towards reproducibility of this work, it would have been preferred for the authors to include the translational velocity and spinneret needle processing parameters in their methodology.

### 2.10. Poly(2-ethyl-2-oxazoline) (PEtOx)

PEtOx belongs to a family of hydrophilic polymers termed the poly(2-oxazolines)s (POx). These polymers are similar to PEO but have a greater degree of tunability and functionalization, which make them ideal candidates for drug delivery applications [33]. In 2014, Hochleitner et al. were the first to explore the major processing parameters for MEW extrusion of this polymer using a custom setup with a proportional integral derivative controller (PID) regulated melt system supplied by a nitrogen gas feeding system and fibers were collected on an aluminum X-Y stage [34]. The authors explored fiber diameter and morphology-based on the processing parameters of temperature (200–220 °C), feeding pressure (1–3 bar), applied voltage (3–7 kV), air gap distance (3–7 mm), and spinneret gauge (23–30 G) while adjusting the translational velocity to be the minimum speed required (200–400 mm/min) to produce a straight fiber. The average fiber diameter ranged from 8 to 138 μm across the explored processing parameters. Of note, the authors observed instances of fiber pulsing, the phenomenon in MEW where mass flow imbalance between the spinneret and collector resulting in prominent oscillations in the fiber’s diameter, in their characterization of PEtOx. The data showed that the greatest fiber pulsing resulted when using the large diameter 23 G spinneret, which posed the greatest mass flow imbalance. Lastly, Hochleitner et al. demonstrated that PEtOx could be readily stacked in grid geometry with scaffolds consisting of 180 layers. Scanning electron micrographs (SEMs) showed that while the fibers were well-formed and aligned, but were unable to be perfectly stacked, which the authors attributed to residual electrostatic charges on the fibers. In conclusion, this work demonstrated that the commercially available polymer PEtOx without modifications can be readily manufactured by MEW to produce high-quality fibers and that the MEW processing parameters methodology outlined in this work was thoroughly detailed for external reproducibility. Furthermore, despite the authors lamenting the inability of the polymer/MEW apparatus to produce perfectly stacked fibers, the ability to create high-quality fibers with defined alignment has significant merit and numerous potential future works for hydrophilic and thermoresponsive tissue engineering scaffolds.

### 2.11. Gelatin

Gelatin is a soluble polypeptide derived from denatured collagen harvested from animals. As it is denatured collagen, gelatin contains numerous arginine-glycine-aspartic (RGD) sequences that modulate cell adhesion making it an ideal non-immunogenic ECM material for tissue engineering [35]. In 2020, Davis et al. were the first to explore the processing parameters for NFES production of gelatin solutions for the potential creation of fibrous structures for musculoskeletal tissue engineering [36]. Towards this, gelatin solutions using porcine-derived, Type A, 300 bloom at concentrations ranging from 450 to 650 mg/mL in acetic acid varying from 60% to 90% were processed by NFES using a custom-built apparatus based on a modified commercially available 3D printer. The major processing parameters gelatin concentration (450–650 mg/mL), acetic acid concentration (60–80%), air gap distance (1–4 mm), spinneret gauge (20–25 G), and translational velocity (15–90 mm/s) were explored using an appropriate applied voltage between 1 and 6 kV and polymer feeding pressure between 0 and 0.4 PSI to produce uniform, cylindrical fibers. Fibers were deposited as 2 layers tall grid scaffolds 20 mm × 20 mm with a fiber spacing of 1 mm. The authors reported that ideally shaped fibers could be tailored between 1.9 and 4.7 μm and that fiber diameters were inversely proportional to gelatin solution concentration within the explored range. The precision of the fiber placement of their custom setup was evaluated by creating grids with equal spacing ranging from 50 to 1000 μm, which the data showed that there was no statistical difference in fiber placement compared to theoretical at spacings of 250 μm and above. In summary, the authors demonstrated that the natural polymer gelatin could be reliably extruded by NFES to produce consistent cylindrical microfibers using their setup and processing parameters. This is in opposition to the current progress with NFES manufacture of collagen, which was unable to reliably produce uniform cylindrical fibers despite being compositionally identical [18]. The biologically relevant binding site on gelatin combined with the ability to custom program scaffold geometry makes this polymer a promising candidate to be used as biomedical scaffolds beyond the two layers demonstrated in this work. Immediate future works entail studying the role of humidity in gelatin fiber formation and further optimizing parameters for a more robust NFES manufacturing process.

### 2.12. Poly(l-Lactic Acid) (PLLA)

PLLA is a biodegraded polyester with bulk mechanical properties widely varying based on average molecular weight and degree of crystallinity. In vivo, PLLA is degraded by hydrolysis of the ester backbone resulting in lactic acid, a biological metabolite [37]. In 2015, Yuan et al. were the first to explore the major processing parameter for NFES processed PLLA to ultimately develop 3D scaffolds for tissue engineering applications [38]. Specifically, they demonstrated that a 5% PLLA solution doped with PEO (80/20, *w*/*w*) in trifluoroethanol (TFE) could reliably create NFES microfibers. The authors evaluate translation speed (100–200 mm/s), polymer flow rate (0.02–0.08 mL/hr), and air gap distance (50–100 mm) while holding the spinneret needle at 22 G and applied voltage at 1 kV/cm constant, which resulted in average fiber diameters ranging from 1.5 to 11.3 µm. They used their optimized parameters to create 1.7 µm fibers, which were stacked for 50 layers to create either a 0°/90° grid and a 0°/45° parallelogram scaffold geometry approximately 100 µm thick. The authors evaluated 3 mm and 5 mm grid spacings and 2 mm parallelogram spacing in which they observed worsening fiber placement fidelity with reduced spacing. They attributed this result due to excess residual electric charges on the fibers repulsing and thus contributing to the bending instability. Surface pore sizes were analyzed using ImageJ to evaluate both optical images and scanning electron micrographs. Based on their two grid and one parallelogram geometry, the authors report a range of tailored pore sizes from 23 ± 10 to 46 ± 17 µm. Subsequently, Yuan et al. cultured human smooth muscle cells (HUASMCs) and seeded them on their 0°/45° geometry scaffolds and TES scaffolds of similar fiber size. Cell proliferation was evaluated on days 1, 4, and 7 and cell infiltration into the scaffolds was evaluated on day 5. The data showed that on days 4 and 7, HUASMCs on NFES constructed scaffolds had statistically greater proliferation with more than double the number of cells on day 7. NFES scaffolds also resulted in greater cell infiltration with DAPI labeled nuclei being measured 72.5 ± 1.7 μm into the scaffold from the seeded edge compared to 16.1 ± 3.8 μm for TES scaffolds. In conclusion, Yuan et al.’s work demonstrated that a PLLA/PEO blend polymer can readily be manufactured by NFES to produced high-quality fibers. These fibers under the evaluated processing parameters resulted in aligned but not perfectly stacked fibers, which allowed for greater cell infiltration compared to traditionally electrospun scaffolds while not resulting in the cells simply falling through. Future work for these scaffolds is anticipated to include use in 3D cell culture for tissue regeneration.

**Figure 4:**
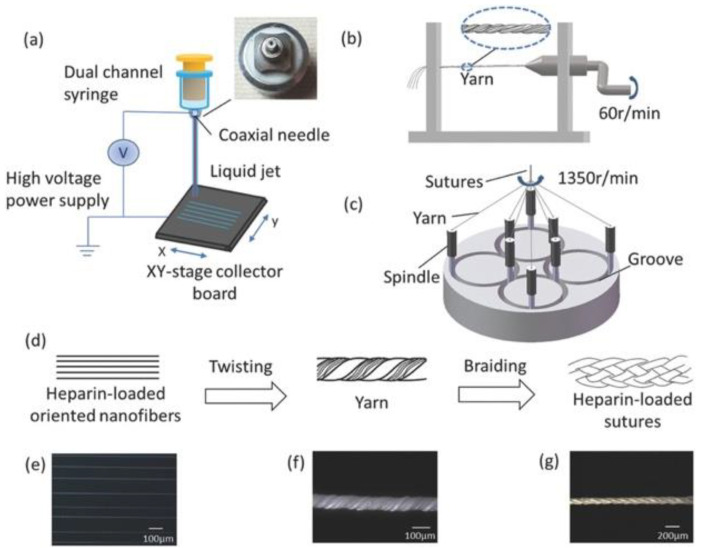
Sketch of suture fabrication. (**a**) Schematic of coaxial NFES; (**b**) schematic of twisting device (ten nanofibers twisted into yarn); (**c**) schematic of knitting machine; and (**d**) sutures: from fibers to suture. Optical microscopy images of (**e**) core–shell fibers (×70), (**f**) yarn (×210), and (**g**) suture (×70). Reproduced with permission from Ye et al., Macromol. Biosci; published by Wiley-VCH GmbH, Weinheim, 2018.

Another example of NFES fabrication of PLLA was in 2018, Ye et al. explored using a complex core and shell setup to create sutures for heparin drug delivery in Achilles tendon rupture to facilitate regeneration [39]. PLLA was dissolved in HFP at a concentration of 16% and subsequently, heparin was dissolved at 0.5%, 0.75%, 1.5%, and 3% to form the shell solution. The core solution was formed by dissolving polyamide (PA) 6 in HFP at a 25% concentration. These polymers were extruded through a dual-channel syringe consisting of a 0.70 mm ID shell nozzle with a 0.24 mm ID core nozzle. Using optimized parameters of 4 mL/h polymer core flow rate, 2 mL/hr polymer shell flow rate, 2.5 kV applied voltage, and 5 mm air gap distance (translational velocity not reported) the authors were able to produce distinct core and shell fibers with diameters of 0.74 µm. The NFES fibers were collected and twist into a yarn followed by braided into sutures, Figure 4a–g. Ye et al. reported that NFES fibers formed into sutured served as a reliable drug delivery platform with 70–90% of the heparin payload being delivered by 14 days. Hemocompatibility was evaluated with platelet-rich plasma from New Zealand rabbits and with the data showing that higher concentrations of loaded heparin resulted in a statistically significant reduction in adhered platelets at 1 h. In vivo evaluation of the NFES fiber sutures was initially performed by implantation in the peritonea of Sprague- Dawley (SD) rat for histocompatibility analysis followed by evaluation of the sutures in an Achilles tendon rupture model. The data showed in the histocompatibility model that the maximum concentration of heparin resulted in less than half the inflammatory cell infiltrate at 2 weeks and an eighth of the number of infiltrates at 4 weeks compared to PLLA alone. The Achilles tendon rupture model after 4 weeks showed the NFES sutures had the same amount of TGF-β1 on immunohistochemistry compared to commercially available sutures, but increasing concentrations of heparin resulted in up to double the TGF-β1 stained per high power field. In conclusion, the authors demonstrated a novel application of harvesting aligned NFES core in shell fibers to form sutures for drug delivery applications. These sutures have significant clinical potential for Achilles tendon rupture and other tendon treatments in future works. Nevertheless, as NFES is a relatively low throughput technique, it may have been better served if the authors used gap electrospinning or touch spinning to achieve uniaxially aligned fibers to form sutures as these methods can produce tight, aligned bundles of fibers at a greater output [40,41].

### 2.13. Polymethyl Methacrylate (PMMA)

PMMA, also known as acrylic, is a transparent thermoplastic that is an organic glass at body temperature with a glass transition temperature ranging from 85 to 165 °C [42]. Biomedically, PMMA is frequently used in orthopedic surgery as bone cement. In 2017, Fattahi et al. were the first to explore NFES manufacturing of PMMA dissolved in nitromethane and fiber written with the first published use of a modified commercial 3D printer as opposed to an X-Y/Z motion stage [43]. This group demonstrated that PMMA fibers with diameters ranging from 1.5 to 4.7 µm based on using a 25 G spinneret needle over the evaluated range of processing parameters of polymer concentration (16–24%), polymer flow rate (50–150 µL/hr), air gap distance (1.6–2.4 mm), applied voltage (1.2–2.4 kV), and translational velocity (10–60 mm/s) could be reliably written in parallel lines, stacked grids, and sinusoidal waves. The authors evaluated the interactions with human mesenchymal stem cells (hMSCs) seeded onto fibers written on poly(2-hydroxyethyl methacrylate) (pHEMA) coated glass slides. After 2 days of culture, the hMSCs actin cytoskeleton was fluorescently stained and quantified for their alignment to the PMMA fibers. The data showed that PMMA microfibers could direct cellular alignment through contact guidance as 95% of the hMSCs aligned with the fibers. Fattahi et al. further demonstrated 3D applications by NFES extruding a grid pattern of PMMA fibers in hMSC loaded collagen gels. Analysis of the gels showed that cells in proximity to the fibers inside the gel reorganize their actin cytoskeleton to align with the PMMA fibers. Therefore, NFES fibers serve to mechanically reinforce the collagen gels and organize cellular alignment in 3D space. In summary, Fattahi et al. detailed the first use of a modified consumer 3D printer for NFES applications. Due to the low throughput of NFES fabrication and the high barrier of entry for many X-Y motion platforms, this work provides a promising solution to ameliorating fiber writing’s throughput limitations. Nevertheless, it would have been ideal if the authors had shown the capacity of PMMA fibers to be stacked to form a handleable scaffold beyond the reported four layers to demonstrate any potential precision limitations of a consumer 3D printer.

### 2.14. Polystyrene (PS)

PS is a non-biodegradable thermoplastic polymer that biomedically is most notably used as tissue culture plastic [44]. In 2012, Xin et al. was the first to explore NFES creation of fibers of 25% PS solutions dissolved in dimethylformamide using a glass pipette with a 0.3 mm ID tip to explore the processing parameters of applied voltage (2.5–6 kV), air gap distance (2–8 cm), and translational velocity (0.1–2.8 m/s) for their effects on fiber bending instability, the main non-axisymmetric instability [45]. The extruded fiber was recorded using a high-speed camera and the fiber patterns were analyzed. They reported that bending instabilities were positively correlated with applied voltage and air gap distance, and negatively correlated with polymer concentration. Increasing the translational velocity resulting in elongating the curling buckling patterns, which turned into wavy patterns, followed by a straight line fiber. These findings are consistent with other NFES published works and are mechanistically explained by charge repulsions on the fiber resulting in bending, fiber mass, and fiber buckling [46,47,48]. In summary, Xin et al. demonstrated as a proof of concept that PS microfibers can be readily NFES. Towards reproducibility, it is unclear if the authors did not report a polymer flow rate or if the applied voltage served to dictate the flow. With their setup, PS resulted in high precise curled and wavy fibers, which can have numerous biomedical applications such as energy storage from their potential spring-like behavior. In future works, it could be of interest to see macro-sized scaffolds with wavy fibers with highly elastic properties.

### 2.15. Poly(Vinyl Alcohol) (PVA) Blends

PVA is a water-soluble synthetic polymer that is frequently used in soft contact lenses and tissue engineering applications [49]. In 2013, Yan et al. explored NFES processing of a blend of PVA and chitosan for tissue engineering scaffold applications [50]. Specifically, 82.5% deacetylated chitosan dissolved in a 10% acetic acid solution and was blended in a 2:1 ratio with 8% PVA solution dissolved in water to be extruded by NFES. A servo motor based custom setup was used to provide the precise relative motion needed for NFES, and it was reported that translational speeds up to 150 mm/s were possible and produced consistent cylindrical chitosan/PVA fibers. The authors evaluated the precision of fiber placement for 200 and 400 µm spacings and reported an average error rate of less than 3.25% from a perfect theoretical fiber placement. In subsequent published work, the authors explored the capacity to create 3D tissue engineering scaffolds with chitosan/PVA fibers by using a hybrid pressure extrusion and NFES approach [51]. An alternating series of grids first consisting of chitosan/PVA polymer layer extruded at a pressure of 0.12 MPa, translational rate of 13 mm/s, and spacing of 1 mm without an applied voltage resulted in large PVA/chitosan struts. The alternating layer using optimized NFES parameters of 2 kV applied voltage, 200 mm/s translational velocities, 1 mm air gap, and 0.1 µL/hr polymer flow rate through a 0.2 mm ID needle, to write chitosan/PVA fibers with an infill spacing of 100 µm. The composite extrusion and NFES chitosan/PVA scaffold resulted in a structure with an average porosity of 55%, and it was mechanically evaluated in compression with a reported modulus of elasticity of 288 MPa and a maximum compressive load of 401 N (no standard deviations reported). In summary, the authors demonstrated a successful proof of concept for their hybrid extrusion/NFES where the extruded chitosan/PVA would serve as the primary load-bearing structure while the NFES chitosan/PVA fibers could potentially interact favorably with cells. Nevertheless, it would have been insightful if the authors explored multiple parameters of their hybrid scaffold geometry to ascertain the tailorability for future scaffold applications. Immediate future works for these PVA/chitosan scaffolds include evaluation of cell viability and proliferation followed by in-depth in vitro analysis.

Another example of PVA blends was in 2020, Cheng et al. NFES manufactured a 10 wt % PVA in water solution emulsified with polytetrafluoroethylene (PTFE) with an unreported amount of boric acid for the creation of tailorable scaffolds [52,53]. PTFE/PVA ratios (4:1–10:1), sintering temperatures (280–430 °C), and scaffold geometries were explored while holding air gap (4 cm), applied voltage (2.85 kV), and polymer flow rate (4.2 µL/min) constant (nozzle dimensions not reported). For an initial evaluation, scaffold grids were fabricated by NFES for 6 layers with side strut lengths of 0.6 mm. After scaffold deposition, sintering was used to remove the PVA leaving only PTFE behind. The data showed that there was a proportional relationship between the degree of crystallinity and sintering temperature between 280 and 380 °C of 91–97.1% with heat shrinkage occurred at 430 °C. Furthermore, the authors reported that varying the PTFE/PVA ratio resulting in an inverse correlation with PTFE crystal size. To demonstrate the tailorability of their PVA/PTFE emulsion and setup, the authors demonstrated the formation of triangle, diamond, square, and hexagon scaffolds geometries with struts diameters ranging between 100 and 400 µm. In conclusion, while classic cylindrical fibers were not formed using this PTFE/PVA bend, the authors demonstrated the first instance of NFES production from an emulsion solution to form highly tailorable scaffolds with potential future applications in cardiovascular medicine.

### 2.16. Polyvinylidene Fluoride (PVDF)

PVDF is a thermoplastic fluoropolymer that is frequently used as a filter membrane, a membrane for blotting techniques, such as a Western blot, sutures, and piezoelectric applications [54]. In 2013, Fuh et al. NFES processed PVDF to investigate the piezoelectric properties of directly written fibers for energy harvesting applications [55]. The authors explored PVDF (M_W_: 534,000 g/mol) dissolved in a 1:1 ratio of N, N-dimethylformamide and acetone with fluorosurfactant at a concentration of 16 wt %. An X-Y stage was used to deposit fibers with an optimized air gap distance of 1.2 mm and 1.4 kV applied voltage (translational velocity and spinneret nozzle not reported) across two copper electrodes. The resulting energy harvesting cells measured 3.5 mm × 1 mm × 2 µm and consisted of a maximum of 20,000 directly written PVDF fibers with diameters ranging from 0.9 to 2.5 µm. The energy harvesting cells were subjected to 0.5% strain at a rate of 10 Hz and which resulted in a peak of 4 V and 75 nA in the forward loaded direction. The NFES PVDF cells were capable of being connected in either series or parallel for scalable energy harvesting. The authors further evaluated the capacity of direct-write PVDF fibers to be implemented in wearable electronics. PVDF fibers were written onto thin copper film and adhered to a woven cotton cloth. The cloth was fixed to a human index finger in which typical finger flexion and extension resulted in a maximum of 0.8 V and 30 nA captured. In summary, this work demonstrates a proof of concept that directly fiber writing of PVDF is an ideal method of creating the β crystalline phase responsible for the piezoelectric properties and the fiber alignment necessary for synergistic energy capture in a similar manner to the polymer PBLG. Future works are anticipated to include wearable electronics that harvest mechanical energy through human motion.

### 2.17. Polyvinylpyrrolidone (PVP)

PVP is a water-soluble polymer that is frequently used as a drug delivery platform and a blood plasma volume expander [56]. In 2019, Guo et al. explored NFES extrusion of PVP dissolved in ethanol to create precise helical fibers for potential large-scale production of morphologically controllable spiral fibers [57]. An X-Y axis platform with a translational speed of 300 mm/s, 30 G needle, a polymer flow rate of 0.4 µL/min at an air gap of 8 mm was used to evaluate 10%, 12%, and 14% PVP solutions at applied voltages between 1.2 and 1.8 kV. The authors reported helically coiled, cylindrical fibers with diameters ranging between 0.9 and 3 µm over the applied voltage range of 1.2–1.4 kV. At higher applied voltages fibers transitioned from helically coiled, cylindrical fibers to straight, wet fibers with inconsistent diameter. Furthermore, greater applied voltages were required to achieve the same ideal helical pattern for increasing polymer concentrations. In summary, this work demonstrated the proof of concept that PVP could be readily NFES fabricated to produce helically written fibers for potential future works in microelectromechanical systems (MEMSs) applications and increase the elasticity of tissue engineering scaffolds. Unfortunately, the range of explored parameters did not result in the ability to directly write uniform, cylindrical straight line fibers.

## 3. Present Limitations

Direct fiber writing demonstrates significant advantages for biomedical scaffold applications through the introduction of another dimension of design tailorability. These advantages are currently not limited to specific subsets of biomedical polymers as there is currently no literature reporting that a polymer fiber cannot be fabricated by NFES/MEW if it could originally be fabricated by TES. We believe that if a Taylor cone can be formed for TES processing, then there should exist a set of parameters that can accommodate direct fiber writing. As a result, a significant percentage of biomedical polymers previously processed to produce TES fibers have to date also successfully manufactured NFES/MEW fibers, Table 1. Nevertheless, outside of MEW fabricated PCL, all other fiber-written polymers were considerably less mature in their development. We believe that improvements to increase development and adoption across the field can be gained by standardization in published setup details, improved fiber write throughput, and increased ease in the creation of complex scaffold geometries.

Standardization in published setup details is vital for the external reproduction of results and will lower the troubleshooting barrier for fiber write adoption by new groups. Many of the works detailed in this review and numerous others fail to completely describe all of the processing parameters needed to reproduce their work. The field of NFES/MEW needs to standardize the reporting of a minimum the main processing parameter of applied voltage/field strength, polymer solution/melt composition, polymer flow rate/driving pressure, relative translational velocity, air gap distance, and spinneret needle geometry. Furthermore, the minor processing parameter of relative translational acceleration, environment temperature, and environment RH should be considered as well. Within these parameters, spinneret geometry is currently only reported as ID, but the length is also vital for reproducibility as it can alter the dynamics of fiber deposition. In 1969 Geoffrey Taylor, whose namesake we get the Taylor cone, derived an equation for the critical voltage required to form a Taylor cone [27]. The parameters involved were spinneret needle ID, needle length, distance from the needle tip to the collector, and liquid surface tension. This theoretically derived equation validated by experiment work with glycerin and water fluids demonstrated that the critical voltage required is inversely proportionally to the length of the spinneret needle. Furthermore, Yang et al. TES fabricated PEO solutions using spinneret needles ranging from 0.5 to 2.5 cm and recorded the dynamics using a high-speed camera imaging at 8000 frames per second. They reported numerous differences, but of note to direct fiber writing they reported that the length of the straight region of the fiber before bending instabilities occurred was inversely proportional to the length of the needle [28]. Finally, Hekmati et al. TES extruded polyamide-6 and explored the effects of spinneret needle length ranging from 10 to 40 mm while holding all other parameters constant [29]. They reported that there were statistically significant differences between the fiber diameters ranging from a 10 mm needle, 155 ± 14 nm, and a 40 mm needle, 286 ± 34 nm. Taken together these results suggest that spinneret needle length should be reported as a major processing parameter for a completely reproducible setup as this knowledge will aid both new and existing fiber write groups.

Beyond needle parameters, the minor processing parameter of relative translational acceleration and RH should be considered. Relative translational acceleration has implications in accuracy and precision for open-loop fiber write systems and fiber lag for all NFES/MEW systems [92,93]. For open-loop systems typical of commercial 3D printers, excessive acceleration for a given mass will result in missed steps from the stepper motor and thus loss of positional accuracy. A closed-loop system that has position encoding can account for these issues and is more frequently implemented in industrial/scientific-grade X-Y/Z motion platforms. For all systems, acceleration in tandem with velocity will affect corning accuracy. Due to the tensile drag force on a fiber depositing onto the collector, acceleration for a given system will vary on how accurately a fiber can be placed while changing vectors. Consequently, this will affect both scaffold dimensional accuracy and to what degree edge effects are present in the final scaffold and therefore should be a considered parameter to explore as well as report.

Another minor processing parameter not frequently reported is RH. It has been extensively demonstrated within TES that RH can affect fiber diameter and morphology. Specifically, Netarati et al. showed that TES extrusion of 18 wt % PCL in 80:20 chloroform: N,N-dimethylformamide solutions at RH values between 5% and 35% resulted in cylindrical, smooth fibers with numerous breakages along the length of the fiber [94]. Alternatively, RH values above 60% resulting in a continuous fiber with rough porosity. For TES extruded from 10% (*w*/*w*) PVA aqueous solutions, Pelipenko et al. demonstrated that for otherwise identical processing conditions an RH of 4% resulted in fiber diameters of 667 ± 83 nm while an RH of 70% resulted in an average diameter of 74 ± 99 nm [95]. Within NFES, Alexander Jr. et al. reported that 250 mg/mL of type 1 collagen in acetic acid was unable to be extruded via NFES for an RH < 50%. As a result, this group used an ETS-5532 environmental chamber to tightly regulate the environmental RH to facilitate the NFES processes [18]. Therefore, this parameter should be reported for more standardized reproducibility and considered as a parameter to be explored for groups having difficulty with consistent NFES extrusion.

Towards improved fiber throughput for NFES/MEW, the reduction in fiber production combined with increased setup complexity compared to TES is one of the greatest barriers to the technique’s adoption by new lab groups and rapid fiber write development by existing groups. Improvements in fiber write throughput are a function of cost, degree of complexity, and desired fiber precision. The first setup decision should be determining melt vs. solution as the complexity and rationale for these fiber write techniques vary. Solution-based NFES manufacturing has a simpler apparatus setup as once a polymer is in solution, a basic syringe pump can to used to supply polymer to the spinneret. Nevertheless, the choice of NFES polymer solvent can be physically and environmentally toxic. As a result, sufficient ventilation must be used, which increases the overall setup complexity. In contrast, MEW manufacturing has a more complex apparatus setup as polymer melt heating and driving pressure must be incorporated into the setup print head and can suffer from fiber pulsing from polymer disposition imbalance [96].

The second decision should be to determine the method of providing precise relative motion between the polymer source and collector. Setups are largely divided into two categories: modified commercial 3D printers or industrial/scientific-grade X-Y/Z motion platforms. Modified 3D printers present the least expensive options to provide relatively precise motion. It is important to note that all commercial 3D printer’s accuracy and precision are not uniform and can be tailored primarily by the quality of the stepper motor and driver, degree of implemented microstepping, degree of step skipping determined by setup conditions, and the radius of the belt drive sprocket. If the degree of accuracy and precision is sufficient for the fiber write design criteria, then modified 3D printers present an excellent low-cost option that can be scaled to use multiple setups in parallel. For example, in our lab, we used two modified commercial 3D printers to improve total NFES fiber output. Alternatively, industrial/scientific-grade X-Y/Z motion platforms are more expensive but allow for greater velocity and torque demands while maintaining typically greater accuracy and precision. As a result, these systems can accommodate faster translational speeds to produce targeted fiber parameters at an increased polymer flow to increase throughput without sacrificing precision.

Lastly, all of these setup considerations can be parallelized by the addition of multiple grouped spinnerets [24,74]. At the cost of increased complexity, using more than one spinneret can greatly increase manufacturing throughput by either in series reducing the time for one scaffold or in parallel by creating more than one scaffold at a time per setup. If the target scaffold geometry has sufficient translational symmetry, then multiple spinnerets can be affixed to one print head in a configuration compatible with the scaffold geometry. This increased throughput is best exemplified by D’Amato et al. who demonstrated a two and six spinneret needle set up to increase the throughput and manufacturing a single tubular scaffold [97]. The cylindrical symmetry of the wind angle geometry allowed for the multiple spinnerets to drastically reduce the manufacturing time for a single scaffold. Alternatively, multiple spinnerets can be affixed with sufficient spacing to one print head such that more than one independent scaffold can be created in parallel per setup.

Towards increased options for scaffold geometry, scaffold geometry can be broken down to fiber geometry and the macro scaffold geometry comprised of that fiber. Beyond throughput, one of the greatest discrepancies between NFES/MEW fabrication and TES fabrication is fiber geometry. TES fiber geometry diameters range typically between 200 nm and 5 µm, while NFES/MEW fiber writing sustained for entire scaffolds is most frequently reported between 2 and 50 µm. Developing a broader range of scaffold fiber diameters particularly in the nanometer range is paramount for the continued future of NFES/MEW as a highly tailorable manufacturing process. These smaller fiber diameters have important implications for recapitulating ECM-sized fibers and for applications that require a greater ratio of surface area to volume. The reduced air gap makes this barrier exceedingly difficult to overcome and will most likely be the result of greatly optimized processing parameters and environmental conditions to achieve consistent nanometer-sized fibers.

One of the greatest limitations to macro scaffold geometry is the ease of programming. The vast majority of geometries published are perfectly stacked fiber grids. While grids are a simple 3D geometry to program and systematically evaluate, it is important to explore more complex architectures. Most relative motion systems use the G-code programming language, which is traditionally generated from a sliced 3D model. These systems were not created with fiber write biomedical scaffolds complexity and fiber spacing in mind to generate G-code movements. In our lab, we elected to manually write the G-code instructions for simple geometries, and for more complex geometries we used the open-source Python library Mecode. This library allows for the generation of G-code at a greater level of abstraction in the more user-friendly python programming language. We believe that specific libraries, slicers, and graphical user interfaces (GUIs) will need to be created specifically for fiber writing to lower the barrier for the creation of more complex architectures [98,99].

## 4. Conclusions and Future Direction

In conclusion, direct fiber writing by NFES and MEW results in an unprecedented increase in 3D scaffold tailorability for biomedical applications. The vast number of biomedical polymers that have been fiber written to date suggest a robust process that gives the user the choice of independent parameters for polymer, geometry, and fiber size. For piezoelectric applications, the ability to created highly ordered fibers in the axis of deformation greatly increases the efficiency of energy capture with non-cytotoxic polymers. For tissue engineering applications as native tissue contains a hierarchy of ECM and cell architecture ranging from randomly distributed to highly organized, having the ability to create highly ordered fibers to recapitulated these complex structures represents the next generation of biomaterials. As the field moves forward, we anticipate advancements in adjacent fields such as modeling and automation to assist in direct writing implementation [80,100,101,102]. Ultimately, barring improvements in fiber throughput, fiber range, and scaffold complexing, the process of fiber writing by NFES and MEW allows for the precise creation of intelligent biomaterials that command the responses of the body forward.

## Figures and Tables

**Table 1:** Collated fiber diameter ranges, fiber write apparatus, and scaffold creation of NFES/MEW processed biomedical polymers.

Polymer	Fiber Diameter (µm)	Stand-Alone Scaffolds	Solution/Melt	Relative Motion	Geometry	Reference
Alginate	1.5–11.3	N	Solution	X-Y Stage	Parallel Fibers	[7]
PBLG	2.5–44	Y	Solution	X-Y, Cylindrical Mandrel	Parallel Fibers	[9,10]
PCL	0.8–150	Y	Both	X-Y Stage, 3D Printer, Cylindrical Mandrel	Perfect Grids, Triangles, Spirals, Imperfect Grids, Words	[12,43,58,59,60,61,62,63,64,65,66,67,68,69,70]
Chitosan	0.3–1.2	Y	Solution	X-Y Stage	Perfect Parallel Lines and Arcs, Grids	[15,16,50,51]
Collagen	1–2	Y	Solution	X-Y Stage	Imperfect Parallel Fibers	[18]
Poly(LLA-ε-CL-AC))	25	Y	Melt	X-Y Stage	Imperfect Grids	[19]
Poly(urea-siloxane)	10.6–17.8	Y	Melt	X-Y Stage	Perfect Grids	[20]
Polyurethane	3	N	Solution	X-Y Stage	Parallel Lines, Grids	[71]
Poly(methylsilsesquioxane)	100	N	Solution	X-Y Stage	Parallel Lines, Grids	[71]
Poly(lactide-block-ethylene glycol-block-lactide)	31	Y	Melt	NA	Perfect and Imperfect Grids	[72]
PDO	3.2–25.3	Y	Solution	3D Printer	Perfect and Imperfect Grids	[23]
PEO	0.05–60	N	Solution	X-Y Stage	Parallel Fibers, Perfect Grids, Words	[4,24,25,46,47,63,73,74,75,76,77,78,79,80,81,82]
PEtOzi	45	Y	Melt	NA	Perfect Grids	[32]
PEtOx	8–138	Y	Melt	X-Y Stage	Imperfect Grids	[34]
Gelatin	1.9–4.7	N	Solution	3D Printer	Perfect Grid	[36]
PLLA	0.7–11.3	Y	Solution	X-Y Stage	Imperfect 45/90° Grids, Braided Fiber	[38,39]
PMMA	1.5–4.7	N	Solution	3D Printer	Perfect 90° Grid	[43]
PS	0.5–1.5	N	Solution	X-Y Stage, Pneumatic 1D Rail	Helical and Straight Fibers	[45,82]
PTFE	100–400	Y	Solution	X-Y Stage	Triangle, Diamond, Grid, and Hexagon Struts	[52,53]
PVDF	0.5–55	Y	Both	X-Y Stage, Cylindrical Mandrel	Parallel Fibers, Perfect Grids, Words	[55,83,84,85,86,87,88,89,90,91]
PVP	0.9–3	N	Solution	X-Y Stage	Imperfect Helical & Parallel Lines	[57,82]

## Data Availability

Not applicable.

## References

[1] Chan B.P., Leong K.W. (2008). Scaffolding in tissue engineering: General approaches and tissue-specific considerations. Eur. Spine J..

[2] Doshi J., Reneker D.H. (1995). Electrospinning Process and Apllications of Electrospun Fibers. J. Electrost..

[3] Hohman M.M., Shin M., Rutledge G., Brenner M.P. (2001). Electrospinning and electrically forced jets. I. Stability theory. Phys. Fluids.

[4] Kameoka J., Orth R., Yang Y., Czaplewski D., Mathers R., Geoffrey C., Craighead H.G. (2003). A scanning tip electrospinning source for deposition of oriented nanofibres. Nanotechnology.

[5] Ding J., Zhang J., Li J., Li D., Xiao C., Xiao H., Yang H., Zhuang X., Chen X. (2019). Electrospun polymer biomaterials. Prog. Polym. Sci..

[6] Lee K.Y., Mooney D.J. (2012). Alginate: Properties and biomedical applications. Prog. Polym. Sci..

[7] Fuh Y.K., Wu Y.C., He Z.Y., Huang Z.M., Hu W.W. (2016). The control of cell orientation using biodegradable alginate fibers fabricated by near-field electrospinning. Mater. Sci. Eng. C Mater. Biol. Appl..

[8] Papadopoulos P., Floudas G. (2004). Self-Assembly and Dynamics of Poly(γ-benzyl-L-glutamate) Peptides. Biomacromolecules.

[9] Pan C.T., Yen C.K., Lin L., Lu Y.S., Li H.W., Huang J., Kuo S.W. (2014). Poly(γ-benzylα,l-glutamate) in Cylindrical Near-Field Electrospinning Fabrication and Analysis of Piezoelectric Fibers. Sens. Mater..

[10] Pan C.-T., Yen C.-K., Lin L., Lu Y.-S., Li H.-W., Huang J.C.-C., Kuo S.-W. (2014). Energy harvesting with piezoelectric poly(γ-benzyl-l-glutamate) fibers prepared through cylindrical near-field electrospinning. RSC Adv..

[11] Woodruff M.A., Hutmacher D.W. (2010). The return of a forgotten polymer—Polycaprolactone in the 21st century. Prog. Polym. Sci..

[12] Delalat B., Harding F., Gundsambuu B., De-Juan-Pardo E.M., Wunner F.M., Wille M.L., Jasieniak M., Malatesta K.A.L., Griesser H.J., Simula A. (2017). 3D printed lattices as an activation and expansion platform for T cell therapy. Biomaterials.

[13] Dubey N., Ferreira J.A., Daghrery A., Aytac Z., Malda J., Bhaduri S.B., Bottino M.C. (2020). Highly tunable bioactive fiber-reinforced hydrogel for guided bone regeneration. Acta Biomater.

[14] Kim I.Y., Seo S.J., Moon H.S., Yoo M.K., Park I.Y., Kim B.C., Cho C.S. (2008). Chitosan and its derivatives for tissue engineering applications. Biotechnol. Adv..

[15] Fuh Y.-K., Chen S., Jang J.S.C. (2012). Direct-write, Well-aligned Chitosan-Poly(ethylene oxide) Nanofibers Deposited via Near-field Electrospinning. J. Macromol. Sci. Part. A.

[16] Fuh Y.K., Chen S.Z., He Z.Y. (2013). Direct-write, highly aligned chitosan-poly(ethylene oxide) nanofiber patterns for cell morphology and spreading control. Nanoscale Res. Lett..

[17] Lee C.H., Singla A., Lee Y. (2001). Biomedical applications of collagen. Int. J. Pharm..

[18] Alexander F.A., Johnson L., Williams K., Packer K. (2019). A Parameter Study for 3D-Printing Organized Nanofibrous Collagen Scaffolds Using Direct-Write Electrospinning. Materials.

[19] Chen F., Hochleitner G., Woodfield T., Groll J., Dalton P.D., Amsden B.G. (2016). Additive Manufacturing of a Photo-Cross-Linkable Polymer via Direct Melt Electrospinning Writing for Producing High Strength Structures. Biomacromolecules.

[20] Hochleitner G., Fursattel E., Giesa R., Groll J., Schmidt H.W., Dalton P.D. (2018). Melt Electrowriting of Thermoplastic Elastomers. Macromol. Rapid Commun..

[21] Ping Ooi C., Cameron R.E. (2002). The hydrolytic degradation of polydioxanone (PDSII) sutures. Part I: Morphological aspects. J. Biomed. Mater. Res..

[22] Ping Ooi C., Cameron R.E. (2002). The hydrolytic degradation of polydioxanone (PDSII) sutures. Part II: Micromechanisms of deformation. J. Biomed. Mater. Res..

[23] King W.E., Gillespie Y., Gilbert K., Bowlin G.L. (2019). Characterization of Polydioxanone in Near-Field Electrospinning. Polymers.

[24] Wang Z., Chen X., Zhang J., Lin Y.-J., Li K., Zeng J., Wu P., He Y., Li Y., Wang H. (2018). Fabrication and evaluation of controllable deposition distance for aligned pattern by multi-nozzle near-field electrospinning. AIP Adv..

[25] Xu G., Wang H., Wang Z., Zhang J., Chen R., Zhu Z., Chen X., Lin Y., Zhao Y., Li J. (2019). Accurate fabrication of aligned nanofibers via a double-nozzle near-field electrospinning. Thermal. Sci..

[26] Hrynevich A., Elci B.S., Haigh J.N., McMaster R., Youssef A., Blum C., Blunk T., Hochleitner G., Groll J., Dalton P.D. (2018). Dimension-Based Design of Melt Electrowritten Scaffolds. Small.

[27] Taylor G. (1969). Electrically driven jets. Proc. R. Soc. Lond. A.

[28] Yang Y., Jia Z., Liu J., Li Q., Hou L., Wang L., Guan Z. (2008). Effect of electric field distribution uniformity on electrospinning. J. Appl. Phys..

[29] Hekmati A., Rashidi A., Ghazisaeidi R., Drean J.Y. (2013). Effect of needle length, electrospinning distance, and solution concentration on morphological properties of polyamide-6 electrospun nanowebs. Text. Res. J..

[30] Drury J.L., Mooney D.J. (2003). Hydrogels for tissue engineering: Scaffold design variables and applications. Biomaterials.

[31] Sundaray B., Subramanian V., Natarajan T.S., Xiang R.-Z., Chang C.-C., Fann W.-S. (2004). Electrospinning of continuous aligned polymer fibers. Appl. Phys. Lett..

[32] Nahm D., Weigl F., Schaefer N., Sancho A., Frank A., Groll J., Villmann C., Schmidt H.-W., Dalton P.D., Luxenhofer R. (2020). A versatile biomaterial ink platform for the melt electrowriting of chemically-crosslinked hydrogels. Mater. Horiz..

[33] De la Rosa V.R., Bauwens E., Monnery B.D., De Geest B.G., Hoogenboom R. (2014). Fast and accurate partial hydrolysis of poly(2-ethyl-2-oxazoline) into tailored linear polyethylenimine copolymers. Polym. Chem..

[34] Hochleitner G., Hümmer J.F., Luxenhofer R., Groll J. (2014). High definition fibrous poly(2-ethyl-2-oxazoline) scaffolds through melt electrospinning writing. Polymer.

[35] Young S., Wong M., Tabata Y., Mikos A.G. (2005). Gelatin as a delivery vehicle for the controlled release of bioactive molecules. J. Control. Release.

[36] Davis Z.G., Hussain A.F., Fisher M.B. (2020). Processing variables of direct-write, near-field electrospinning impact size and morphology of gelatin fibers. bioRxiv.

[37] Farah S., Anderson D.G., Langer R. (2016). Physical and mechanical properties of PLA, and their functions in widespread applications-A comprehensive review. Adv. Drug Deliv. Rev..

[38] Yuan H., Zhou Q., Li B., Bao M., Lou X., Zhang Y. (2015). Direct printing of patterned three-dimensional ultrafine fibrous scaffolds by stable jet electrospinning for cellular ingrowth. Biofabrication.

[39] Ye Y.J., Zhou Y.Q., Jing Z.Y., Liu Y.Y., Yin D.C. (2018). Electrospun Heparin-Loaded Core-Shell Nanofiber Sutures for Achilles Tendon Regeneration In Vivo. Macromol. Biosci..

[40] Jha B.S., Colello R.J., Bowman J.R., Sell S.A., Lee K.D., Bigbee J.W., Bowlin G.L., Chow W.N., Mathern B.E., Simpson D.G. (2011). Two pole air gap electrospinning: Fabrication of highly aligned, three-dimensional scaffolds for nerve reconstruction. Acta Biomater..

[41] Tokarev A., Asheghali D., Griffiths I.M., Trotsenko O., Gruzd A., Lin X., Stone H.A., Minko S. (2015). Touch- and Brush-Spinning of Nanofibers. Adv. Mater..

[42] Ali U., Karim K.J.B.A., Buang N.A. (2015). A Review of the Properties and Applications of Poly (Methyl Methacrylate) (PMMA). Polymer. Rev..

[43] Fattahi P., Dover J.T., Brown J.L. (2017). 3D Near-Field Electrospinning of Biomaterial Microfibers with Potential for Blended Microfiber-Cell-Loaded Gel Composite Structures. Adv. Healthc. Mater..

[44] Lerman M.J., Lembong J., Muramoto S., Gillen G., Fisher J.P. (2018). The Evolution of Polystyrene as a Cell Culture Material. Tissue Eng. Part. B Rev..

[45] Xin Y., Reneker D.H. (2012). Hierarchical polystyrene patterns produced by electrospinning. Polymer.

[46] Sun D., Chang C., Li S., Lin L. (2006). Near-Field Electrospinning. Nano Lett..

[47] Chang C., Limkrailassiri K., Lin L. (2008). Continuous near-field electrospinning for large area deposition of orderly nanofiber patterns. Appl. Phys. Lett..

[48] Zheng G., Li W., Wang X., Wu D., Sun D., Lin L. (2010). Precision deposition of a nanofibre by near-field electrospinning. J. Phys. D Appl. Phys..

[49] Baker M.I., Walsh S.P., Schwartz Z., Boyan B.D. (2012). A review of polyvinyl alcohol and its uses in cartilage and orthopedic applications. J. Biomed. Mater. Res. B Appl. Biomater..

[50] Yan F.C.H., Zheng L., Chen W., Liu Y., Hu Q. (2013). The Controllable PVA-Chitosan Fiber Prepared by the Near-field Electro Spinning for Tissue Engineering. Adv. J. Food Sci. Technol..

[51] Yan F., Liu Y., Chen H., Zhang F., Zheng L., Hu Q. (2014). A multi-scale controlled tissue engineering scaffold prepared by 3D printing and NFES technology. AIP Adv..

[52] Cheng J., Huang Q., Huang Y., Luo W., Hu Q., Xiao C. (2020). Study on a novel PTFE membrane with regular geometric pore structures fabricated by near-field electrospinning, and its applications. J. Membr. Sci..

[53] Cheng J., Huang Q., Huang Y., Yu S., Xiao C., Hu Q. (2020). Pore structure design of NFES PTFE membrane for membrane emulsification. J. Membr. Sci..

[54] Laroche G., Marois Y., Guidoin R., King M.W., Martin L., How T., Douville Y. (1995). Polyvinylidene fluoride (PVDF) as a biomaterial: From polymeric raw material to monofilament vascular suture. J. Biomed. Mater. Res..

[55] Fuh Y.K., Ye J.C., Chen P.C., Ho H.C., Huang Z.M. (2015). Hybrid Energy Harvester Consisting of Piezoelectric Fibers with Largely Enhanced 20 V for Wearable and Muscle-Driven Applications. ACS Appl. Mater. Interfaces.

[56] Teodorescu M., Bercea M. (2015). Poly(vinylpyrrolidone)–A Versatile Polymer for Biomedical and Beyond Medical Applications. Polymer-Plast. Technol. Eng..

[57] Guo K., Zhang H.-D., Ma X.-S., Zhu J.-W., Long Y.-Z. (2018). Preparation of arrayed helical micro/nanofibers by near-field electrospinning. Mater. Res. Express.

[58] Zhang Z., Jorgensen M.L., Wang Z., Amagat J., Wang Y., Li Q., Dong M., Chen M. (2020). 3D anisotropic photocatalytic architectures as bioactive nerve guidance conduits for peripheral neural regeneration. Biomaterials.

[59] Jungst T., Pennings I., Schmitz M., Rosenberg A.J.W.P., Groll J., Gawlitta D. (2019). Heterotypic Scaffold Design Orchestrates Primary Cell Organization and Phenotypes in Cocultured Small Diameter Vascular Grafts. Adv. Funct. Mater..

[60] Schaefer N., Janzen D., Bakirci E., Hrynevich A., Dalton P.D., Villmann C. (2019). 3D Electrophysiological Measurements on Cells Embedded within Fiber-Reinforced Matrigel. Adv. Healthc. Mater..

[61] Hammerl A., Diaz Cano C.E., De-Juan-Pardo E.M., van Griensven M., Poh P.S.P. (2019). A Growth Factor-Free Co-Culture System of Osteoblasts and Peripheral Blood Mononuclear Cells for the Evaluation of the Osteogenesis Potential of Melt-Electrowritten Polycaprolactone Scaffolds. Int. J. Mol. Sci..

[62] Hochleitner G., Jungst T., Brown T.D., Hahn K., Moseke C., Jakob F., Dalton P.D., Groll J. (2015). Additive manufacturing of scaffolds with sub-micron filaments via melt electrospinning writing. Biofabrication.

[63] Abbasi N., Abdal-hay A., Hamlet S., Graham E., Ivanovski S. (2019). Effects of Gradient and Offset Architectures on the Mechanical and Biological Properties of 3-D Melt Electrowritten (MEW) Scaffolds. ACS Biomater. Sci. Eng..

[64] He F.L., Li D.W., He J., Liu Y.Y., Ahmad F., Liu Y.L., Deng X., Ye Y.J., Yin D.C. (2018). A novel layer-structured scaffold with large pore sizes suitable for 3D cell culture prepared by near-field electrospinning. Mater Sci. Eng. C Mater Biol. Appl..

[65] Farrugia B.L., Brown T.D., Upton Z., Hutmacher D.W., Dalton P.D., Dargaville T.R. (2013). Dermal fibroblast infiltration of poly(ε-caprolactone) scaffolds fabricated by melt electrospinning in a direct writing mode. Biofabrication.

[66] Brown T.D., Slotosch A., Thibaudeau L., Taubenberger A., Loessner D., Vaquette C., Dalton P.D., Hutmacher D.W. (2012). Design and fabrication of tubular scaffolds via direct writing in a melt electrospinning mode. Biointerphases.

[67] Liashenko I., Hrynevich A., Dalton P.D. (2020). Designing Outside the Box: Unlocking the Geometric Freedom of Melt Electrowriting using Microscale Layer Shifting. Adv. Mater..

[68] Eichholz K.F., Hoey D.A. (2018). Mediating human stem cell behaviour via defined fibrous architectures by melt electrospinning writing. Acta Biomater..

[69] Saidy N.T., Shabab T., Bas O., Rojas-Gonzalez D.M., Menne M., Henry T., Hutmacher D.W., Mela P., De-Juan-Pardo E.M. (2020). Melt Electrowriting of Complex 3D Anatomically Relevant Scaffolds. Front Bioeng. Biotechnol..

[70] Castilho M., van Mil A., Maher M., Metz C.H.G., Hochleitner G., Groll J., Doevendans P.A., Ito K., Sluijter J.P.G., Malda J. (2018). Melt Electrowriting Allows Tailored Microstructural and Mechanical Design of Scaffolds to Advance Functional Human Myocardial Tissue Formation. Adv. Funct. Mater..

[71] Ahmad Z., Rasekh M., Edirisinghe M. (2010). Electrohydrodynamic Direct Writing of Biomedical Polymers and Composites. Macromol. Mater. Eng..

[72] Hochleitner G., Kessler M., Schmitz M., Boccaccini A.R., Teßmar J., Groll J. (2017). Melt electrospinning writing of defined scaffolds using polylactide-poly(ethylene glycol) blends with 45S5 bioactive glass particles. Mater. Lett..

[73] Bisht G.S., Canton G., Mirsepassi A., Kulinsky L., Oh S., Dunn-Rankin D., Madou M.J. (2011). Controlled continuous patterning of polymeric nanofibers on three-dimensional substrates using low-voltage near-field electrospinning. Nano Lett..

[74] Wang H., Li M., Huang S., Zheng J., Chen X., Chen X., Zhu Z. (2014). Deposition characteristics of the double nozzles near-field electrospinning. Appl. Phys. A.

[75] Shin D., Choi S., Kim J., Regmi A., Chang J. (2020). Direct-Printing of Functional Nanofibers on 3D Surfaces Using Self-Aligning Nanojet in Near-Field Electrospinning. Adv. Mater. Technol..

[76] Wang H., Zheng G., Li W., Wang X., Sun D. (2011). Direct-writing organic three-dimensional nanofibrous structure. Appl. Phys. A.

[77] Zheng J., Zhang K., Jiang J., He G., Xu L., Liu Y., Liu J., Wu D., Zheng G. (2016). Electrohydrodynamic direct-writing orderly pattern with sheath gas focusing. AIP Adv..

[78] Huang Y.Y., Terentjev E.M., Oppenheim T., Lacour S.P., Welland M.E. (2012). Fabrication and electromechanical characterization of near-field electrospun composite fibers. Nanotechnology.

[79] Zhu Z., Chen X., Huang S., Du Z., zeng J., Liao W., Fang F., Peng D., Wang H. (2015). The process of wavy fiber deposition via auxiliary electrodes in near-field electrospinning. Appl. Phys. A.

[80] Wang H., Huang S., Liang F., Wu P., Li M., Lin S., Chen X. (2015). Research on Multinozzle Near-Field Electrospinning Patterned Deposition. J. Nanomater..

[81] Lee M., Kim H.Y. (2014). Toward nanoscale three-dimensional printing: nanowalls built of electrospun nanofibers. Langmuir.

[82] Li X., Li Z., Wang L., Ma G., Meng F., Pritchard R.H., Gill E.L., Liu Y., Huang Y.Y. (2016). Low-Voltage Continuous Electrospinning Patterning. ACS Appl. Mater. Interfaces.

[83] Pan C.-T., Chang C.-C., Yang Y.-S., Yen C.-K., Kao Y.-H., Shiue Y.-L. (2020). Development of MMG sensors using PVDF piezoelectric electrospinning for lower limb rehabilitation exoskeleton. Sens. Actuators A Phys..

[84] Luo G., Teh K.S., Liu Y., Zang X., Wen Z., Lin L. (2015). Direct-Write, Self-Aligned Electrospinning on Paper for Controllable Fabrication of Three-Dimensional Structures. ACS Appl. Mater. Interfaces.

[85] Florczak S., Lorson T., Zheng T., Mrlik M., Hutmacher D.W., Higgins M.J., Luxenhofer R., Dalton P.D. (2019). Melt electrowriting of electroactive poly(vinylidene difluoride) fibers. Polym. Int..

[86] Hoe Z.Y., Chang C.C., Chen J.J., Yen C.K., Wang S.Y., Kao Y.H., Li W.M., Chen W.F., Pan C.T. (2020). Enhancement of PVDF Sensing Characteristics by Retooling the Near-Field Direct-Write Electrospinning System. Sensors.

[87] Fuh Y.-K., Chen S.-Y., Ye J.-C. (2013). Massively parallel aligned microfibers-based harvester deposited via in situ, oriented poled near-field electrospinning. Appl. Phys. Lett..

[88] Duan Y., Huang Y., Yin Z., Bu N., Dong W. (2014). Non-wrinkled, highly stretchable piezoelectric devices by electrohydrodynamic direct-writing. Nanoscale.

[89] Kim J., Maeng B., Park J. (2018). Characterization of 3D electrospinning on inkjet printed conductive pattern on paper. Micro Nano Syst. Lett..

[90] Chang C., Tran V.H., Wang J., Fuh Y.K., Lin L. (2010). Direct-write piezoelectric polymeric nanogenerator with high energy conversion efficiency. Nano Lett..

[91] Liu Z.H., Pan C.T., Lin L.W., Lai H.W. (2013). Piezoelectric properties of PVDF/MWCNT nanofiber using near-field electrospinning. Sens. Actuators A Phys..

[92] Weiss B., Storti D., Ganter M. (2015). Low-cost closed-loop control of a 3D printer gantry. Rapid Prototyp. J..

[93] Jin Y., Gao Q., Xie C., Li G., Du J., Fu J., He Y. (2020). Fabrication of heterogeneous scaffolds using melt electrospinningwriting: Design and optimization. Mater. Des..

[94] Nezarati R.M., Eifert M.B., Cosgriff-Hernandez E. (2013). Effects of humidity and solution viscosity on electrospun fiber morphology. Tissue Eng. Part C Methods.

[95] Pelipenko J., Kristl J., Jankovic B., Baumgartner S., Kocbek P. (2013). The impact of relative humidity during electrospinning on the morphology and mechanical properties of nanofibers. Int. J. Pharm..

[96] Hochleitner G., Youssef A., Hrynevich A., Haigh J.N., Jungst T., Groll J., Dalton P.D. (2016). Fibre pulsing during melt electrospinning writing. Bio. Nano Mater..

[97] D’Amato A.R., Ding X., Wang Y. (2021). Using Solution Electrowriting to Control the Properties of Tubular Fibrous Conduits. ACS Biomater. Sci. Eng..

[98] Paxton N.C., Lanaro M., Bo A., Crooks N., Ross M.T., Green N., Tetsworth K., Allenby M.C., Gu Y., Wong C.S. (2020). Design tools for patient specific and highly controlled melt electrowritten scaffolds. J. Mech. Behav. Biomed. Mater.

[99] Bisht G., Nesterenko S., Kulinsky L., Madou M. (2012). A computer-controlled near-field electrospinning setup and its graphic user interface for precision patterning of functional nanofibers on 2D and 3D substrates. J. Lab. Autom..

[100] Xiao B., Wang W., Ding Y., Zhang X., Long G., Fan J., Chen H., Deng L. (2019). A novel fractal solution for permeability and Kozeny-Carman constant of fibrous porous media made up of solid particles and porous fibers. Powder Technol..

[101] Huang Y., Duan Y., Ding Y., Bu N., Pan Y., Lu N., Yin Z. (2014). Versatile, kinetically controlled, high precision electrohydrodynamic writing of micro/nanofibers. Sci. Rep..

[102] Duan Y., Huang Y., Yin Z. (2015). Competing buckling of micro/nanowires on compliant substrates. J. Phys. D Appl. Phys..

